# Anti-*Plasmodium falciparum* invasion ligand antibodies in a low malaria transmission region, Loreto, Peru

**DOI:** 10.1186/1475-2875-11-361

**Published:** 2012-10-30

**Authors:** Elizabeth Villasis, Mary Lopez-Perez, Katherine Torres, Dionicia Gamboa, Victor Neyra, Jorge Bendezu, Nancy Tricoche, Cheryl Lobo, Joseph M Vinetz, Sara Lustigman

**Affiliations:** 1 Malaria Laboratory, Instituto de Medicina Tropical “Alexander von Humboldt”, Universidad Peruana Cayetano Heredia, Lima, Peru; 2 Molecular Parasitology, Lindsley F. Kimball Research Institute, New York Blood Center, NYC, New York, NY, USA; 3 Departamento de Ciencias Celulares y Moleculares, Facultad de Ciencias y Filosofia, Universidad Peruana Cayetano Heredia, Lima, Peru; 4 Blood-Borne Parasites, Lindsley F. Kimball Research Institute, New York Blood Center, NYC, New York, NY, USA; 5 Division of Infectious Diseases, Department of Medicine, University of California San Diego, La Jolla, CA, USA

**Keywords:** Antibodies, Invasion, *Plasmodium falciparum*, Malaria, Peru

## Abstract

**Background:**

Erythrocyte invasion by *Plasmodium falciparum* is a complex process that involves two families; Erythrocyte Binding-Like (EBL) and the Reticulocyte Binding-Like (PfRh) proteins. Antibodies that inhibit merozoite attachment and invasion are believed to be important in mediating naturally acquired immunity and immunity generated by parasite blood stage vaccine candidates. The hypotheses tested in this study were 1) that antibody responses against specific *P. falciparum* invasion ligands (EBL and PfRh) differ between symptomatic and asymptomatic individuals living in the low-transmission region of the Peruvian Amazon and 2), such antibody responses might have an association, either direct or indirect, with clinical immunity observed in asymptomatically parasitaemic individuals.

**Methods:**

ELISA was used to assess antibody responses (IgG, IgG1 and IgG3) against recombinant *P. falciparum* invasion ligands of the EBL (EBA-175, EBA-181, EBA-140) and PfRh families (PfRh1, PfRh2a, PfRh2b, PfRh4 and PfRh5) in 45 individuals infected with *P. falciparum* from Peruvian Amazon. Individuals were classified as having symptomatic malaria (N=37) or asymptomatic infection (N=8).

**Results:**

Antibody responses against both EBL and PfRh family proteins were significantly higher in asymptomatic compared to symptomatic individuals, demonstrating an association with clinical immunity. Significant differences in the total IgG responses were observed with EBA-175, EBA-181, PfRh2b, and MSP1_19_ (as a control). IgG1 responses against EBA-181, PfRh2a and PfRh2b were significantly higher in the asymptomatic individuals. Total IgG antibody responses against PfRh1, PfRh2a, PfRh2b, PfRh5, EBA-175, EBA-181 and MSP1_19_ proteins were negatively correlated with level of parasitaemia. IgG1 responses against EBA-181, PfRh2a and PfRh2b and IgG3 response for PfRh2a were also negatively correlated with parasitaemia.

**Conclusions:**

These data suggest that falciparum malaria patients who develop clinical immunity (asymptomatic parasitaemia) in a low transmission setting such as the Peruvian Amazon have antibody responses to defined *P. falciparum* invasion ligand proteins higher than those found in symptomatic (non-immune) patients. While these findings will have to be confirmed by larger studies, these results are consistent with a potential role for one or more of these invasion ligands as a component of an anti-*P. falciparum* vaccine in low-transmission malaria-endemic regions.

## Background

Erythrocyte invasion by *Plasmodium falciparum* is a complex process including attachment, reorientation, penetration, and formation of a parasitophorous vacuole. Several merozoite proteins that have a role during the initial steps of attachment and invasion have been extensively studied, including members of the Merozoite Surface Protein family (MSP), AMA-1, Erythrocyte Binding-Like proteins (EBL: EBA-175, EBA-181, EBA-140 and EBL-1), and the Reticulocyte Binding-Like or Reticulocyte Homologue proteins (RBL or PfRh: PfRh1, PfRh2a, PfRh2b, PfRh4 and PfRh5) [1]. Many of the invasion ligands are currently being evaluated or developed as candidate vaccine antigens for inclusion in an anti-erythrocytic-stage malaria vaccine [2]. Antibodies that inhibit merozoite attachment and invasion, and thus subsequent development and propagation within the red blood cells (RBC), are believed to be important in mediating naturally acquired immunity as well as immunity generated by parasite blood stage vaccine candidates [3]. Moreover, the cytophilic IgG1 and IgG3 antibody isotype subclasses have been reported to be associated with protective responses generated against invasion ligands [4-6], by enabling the activation of complement and antibody-dependent phagocytosis and consequently parasite clearance [7]. However, it remains unclear which merozoite invasion ligand antigens might be the most important targets of naturally acquired clinical immunity, and whether the importance of such antigens are of regional specificity or globally relevance [2].

Malaria in the Amazonian region is hypoendemic and characterized by a low transmission [8]. The malaria infections are most commonly caused by *Plasmodium vivax*, but *P. falciparum* is still responsible for the major cases of severe malaria, and these infections continue to persist even though control measures are in place [9]. Previous studies in this region have demonstrated that clinical immunity to malaria is manifested by the presence of individuals with asymptomatic parasitaemia, which is not infrequent [8,10]. Importantly, asymptomatic parasitaemia has major implications for public health, particularly in maintaining transmission including the introduction or reintroduction of parasites in endemic regions that stopped having malaria. Understanding the immune mechanisms by which infected humans control parasitaemia in the absence of symptoms has important implications for developing anti-malarial vaccine strategies [10]. In individuals living in areas of intense *P. falciparum* transmission clinical immunity to symptomatic malaria is thought to be acquired only after repeated exposure [2]. In contrast, studies have demonstrated in Indonesia and in Amazonia that acquisition of clinical immunity can be rapid (within two years), especially in adults, and may require few infections [9-15]. This observation clearly indicates that non-sterilizing but effective clinical anti-malarial immunity develops in low transmission regions [9].

Given the epidemiological observations indicating clinical immunity against *P. falciparum*, this study aimed to test the hypothesis that antibody responses against *P. falciparum* invasion ligands belonging to both EBL and PfRh protein families might differ between symptomatic (Sym) and asymptomatic (Asy) individuals living in the low-transmission region of the Peruvian Amazon, and hence potentially contributing to explaining mechanisms of clinical immunity observed in the Asy individuals. Recombinant *P. falciparum* proteins corresponding to the known EBL and PfRh invasion ligands were used to determine the total IgG and IgG isotype-specific antibody responses in both study groups.

## Methods

### Study population

This study was approved by the Universidad Peruana Cayetano Heredia Institutional Review Board (Comite de Etica) in Lima, Peru, and by the New York Blood Center’s IRB (protocol #415). Informed consent was obtained from each adult individual or from the parents or guardians of children less than 18 years of age. The cross-sectional study took place in the Peruvian Amazon region of Loreto in 2008–2010.

A total of 45 plasma samples from individuals infected with *P. falciparum* were collected over the period of 2008–2010 from 14 different communities in Loreto. The individuals were classified into two groups: 37 individuals with symptomatic malaria (16 females and 21 males; median age = 29; range 9–63 years) and eight individuals with asymptomatic infection (one female and seven males; median age = 26; range 12–43 years). Asymptomatic individuals were identified by active case detection after carrying out a malaria diagnostic survey in communities that reported a malaria outbreak. Passive case detection was used to enroll symptomatic individuals who attended the local Health Care Center for diagnosis and treatment and were diagnosed with *P. falciparum* infection by light microscopy. The microscopic diagnosis in all individuals enrolled was confirmed as *P. falciparum* single infection by real time PCR [16]. Individuals were classified as Asy if they did not have any signs or symptoms related to malaria [eg. fever (temperature >37°C), chills, sweating, headache or any other kind of symptoms that the subject interpreted as indicating malaria but were positive by blood smear for *P. falciparum*. Individuals were excluded if they used anti-malarial medication over the two weeks before enrollment and sample collection. No follow up was done. Blood samples were collected before all individuals were treated for malaria with mefloquine/artesunate following the National Drug Policy Guidelines of the Ministry of Health of Peru.

### Proteins used in the ELISA assays

The following recombinant EBL and PfRh proteins corresponding to the *P. falciparum* 3D7 laboratory strain were used for ELISA. The C-terminal region of EBA-181 (aa 1235–1345), PfRh1 (aa 1318–1574), PfRh2a (aa 2813–2933), PfRh2b (aa 2735–3133) and PfRh5 (aa 31–174) were expressed in *Escherichia coli* BL21. PfRh2b and PfRh5 were expressed as fusion proteins with glutathione S-transferase (GST) using the pGEX 4T-3 expression vector (Amersham Biotech, NJ). The *E. coli* expressed PfRh4 (aa 1160–1370) protein was a kind gift from Deepak Gaur (ICGEB; New Delhi, India) and Louis Miller (NIH, USA) [17]. Fragments corresponding to the region II of EBA-175 (aa 145–760) and EBA-140 (aa 141–756) were expressed in *Pichia pastoris* and were a kind gift from David Narum and Louis Miller (NIH, USA) [5]. The recombinant protein corresponding to the 19-kDa processed fragment of MSP-1 (MSP1_19_; MRA-55, MR4, ATCC Manassas Virginia) was also used as a control [18].

### IgG responses against *P. falciparum* invasion ligands as determined by ELISA

Peruvian plasma samples were analyzed for total IgG, IgG1 and IgG3 reactivity to the recombinant EBL and PfRh ligands by ELISA as previously described [19]. Plasma samples were pre-incubated with *E. coli* extract prior to dilution and incubation with the bound antigen to remove antibodies to potential *E. coli* contaminants in the recombinant protein preparations. Briefly, 96-well ELISA plates (Costar, Corning Life Sciences, Corning, NY) were coated with 1 μg/mL of the recombinant protein (0.5 μg/mL for PfRh4) in 0.05 M carbonate buffer, pH 9.6 overnight at 4°C. The next day, plates were washed 6 times with phosphate-buffered saline (PBS) containing 0.05% Tween 20 (PBST) and blocked for 1.5-2 h with blocking buffer (3% BSA in PBST) at 37°C. Plates were washed with PBST and test and control plasma samples (1:200) diluted in blocking buffer were added into each well in duplicate for 2 h at 37°C. The plates were then washed 6 times with PBST and the bound IgG antibodies were detected after 1 h of incubation at 37°C using HRP-conjugated goat anti-human (1:1,000) IgG (H+L; Kirkegaard and Perry Laboratories, Inc., Gaithersburg, MD). IgG1 and IgG3 antibodies were detected after incubation for 1 hr at 37°C with mouse monoclonal antibodies against human IgG1 or IgG3 (Hybridoma Reagent Laboratory, Baltimore, MD) diluted 1:1,000 in blocking buffer, followed by HRP-conjugated anti-mouse (1:1250). After 6X washing with PBST, bound antibodies were detected by adding the SureBlue tetramethyl benzidine (TMB) substrate (Kirkegaard and Perry Laboratories, Inc., Gaithersburg, MD). The reaction was stopped by the addition of 2N H_2_SO_4_, and the Optical Density (OD) at 450 nm was measured using a SpectraMax 190 ELISA Reader (Molecular Devices, Sunnyvale, CA). For the GST-fusion PfRh2b and PfRh5 proteins, each plasma sample was also tested against recombinant GST as a negative control and the corresponding absorbance was deducted from all test values for the fusion proteins. The negative control human serum was composed of a pool of sera from five healthy adult volunteers living in USA who had never been exposed to malaria. Positive responders were defined as those that gave an OD value greater than the mean plus three standard deviations (SD) of the reactivity observed with negative control serum (cut-off value).

### Statistical analysis

Fisher’s exact test (2x2 contingency tables) was used to compare differences in the seroprevalence between groups. The Mann–Whitney test was used to compare medians between different groups. Correlations between antibody response (as indicated by OD) and parasitaemia or age were examined using Spearman’s test. A *p* value < 0.05 was considered statistically significant. Statistical analyses were performed using GraphPad Prism software (version 5.01; GraphPad Software Inc., San Diego, CA, USA).

## Results

### Differential antibody responses in asymptomatic *vs* symptomatic individuals

Two groups of individuals were studied; individuals with symptomatic malaria (Sym, N =37) and individuals with asymptomatic infection (Asy, N=8). There were no significant differences in the sex distribution or median of age between the groups. Parasitaemia was lower in the Asy (median 519 parasites/μL; range 24–2874 parasites/μL) than in the Sym individuals (median 7056 parasites/μL; range 239–61,185 parasites/μL) (*p* < 0.0001).

**Figure 1:**
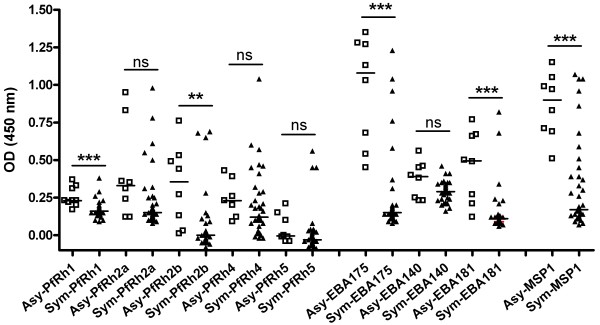
**Total IgG responses to EBL and PfRh ligands in Symptomatic and Asymptomatic Peruvian individuals.** Comparison between the total IgG responses (as indicated by optical density, OD) in asymptomatic (Asy) and symptomatic (Sym) Peruvian individuals. Median values are indicated by horizontal bars. Abbreviations: Asy, open symbols; Sym, close symbols; ns, not significant; ** *p* < 0.01; *** *p* < 0.001.

Samples were tested against a comprehensive panel of known EBL and PfRh ligands except EBL-1; a majority of the Peruvian *P. falciparum* field isolates do not express EBL-1 as the gene in these parasites contained a five-thymidine insertion that results in premature translational termination and lack of protein expression [Lopez-Perez M *et al.*, manuscript in preparation]. Antibodies against MSP1_19,_ an immunogenic merozoite protein targeted as a vaccine protein, were measured for comparison. The percentage of responders against this antigen was 100% in Asy *vs*. 54% in the Sym (Table 1). Based on the total IgG responses against the EBL and PfRh invasion ligands, the percentage of responders in the Asy *vs*. the Sym group varied for some. In general, a higher seroprevalence was observed in Asy individuals against EBA-175, EBA-181, PfRh1, PfRh2b and PfRh4 than in Sym individuals. The percentage of responders against PfRh2a was similar (75%). Notably, there were fewer responders in both groups against EBA-140 (25% and 5.4%) and PfRh5 (25% and 8.1%) (Table 1). When the seroprevalence between both groups for each invasion ligand was compared, it appeared that the percentage of responders in the Asy individuals was significantly higher than in the Sym group only against EBA-175 (*p =* 0.0006), EBA-181 (*p =* 0.0009) and PfRh2b (*p =* 0.007) as well as against the control merozoite protein, MSP1_19_ (*p =* 0.0171). Significantly, a higher total IgG response, as indicated by the OD value, was observed in Asy than Sym individuals for EBA-175 (median OD 1.08 *vs.* 0.15; *p* = 0.0001), EBA-181 (median OD 0.50 *vs.* 0.11; *p* = 0.0004), PfRh1 (median OD 0.23 *vs.* 0.16; *p* = 0.0006) and PfRh2b (median OD 0.36 *vs.* 0.01; *p* = 0.0022) as well as for MSP1_19_ (median OD 0.90 *vs.* 0.17; *p* = 0.0005) (Figure 1).

**Table 1:** Percentages of individuals within the Asymptomatic (Asy) and Symptomatic (Sym) malaria groups responding (total IgG) to EBL and PfRh ligands

**Ligand**	**PfRh1**	**PfRh2a**	**PfRh2b**	**PfRh4**	**PfRh5**	**EBA- 175**	**EBA- 140**	**EBA-181**	**MSP-1**_**19**_
Asy	100%	75%	75%	71%	25%	100%	25%	88%	100%
Seropositive	(8/8)	(6/8)	(6/8)	(5/7)	(2/8)	(8/8)	(2/8)	(7/8)	(8/8)
Sym	86.5%	75.7%	21.6%	47%	8.1%	32. 4%	5.4%	21.6%	54%
Seropositive	(32/37)	(28/37)	(8/37)	(17/36)	(3/37)	(12/37)	(2/37)	(8/37)	(20/37)
Cut-off value	0.13	0.12	0.1	0.1	0.16	0.16	0.41	0.12	0.16
*p* (Fisher Test)	ns	ns	0.007	ns	ns	0.0006	ns	0.0009	0.0171

### Total IgG and isotype responses against some of the invasion ligands are also associated with reduced parasitaemia

**Figure 2:**
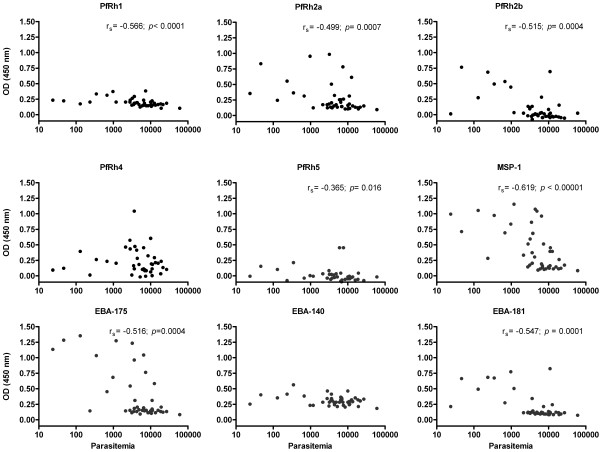
**Total IgG response to EBL and PfRh ligands in Symptomatic and Asymptomatic malaria infected Peruvian individuals in relationship with their parasitaemia.** The parasitaemia was determined by light microscopy and expressed as parasites/μL. Spearman correlation coefficient, abbreviated r_s_*,* and *p* value are shown.

As Asy individuals had significantly lower parasitaemia than Sym, ELISA was used to determine whether IgG levels, as indicated by optical density, were also negatively associated with parasitaemia (parasites/μL). A significant negative correlation was observed between the total IgG levels and parasitaemia for the following invasion ligands: PfRh1 (*p* < 0.0001, r_s_ = −0.565), PfRh2a (*p* = 0.007, r_s_ = −0.499), PfRh2b (*p* = 0.0004, r_s_ = −0.515), PfRh5 (*p* = 0.016, r_s_ = −0.364), EBA-175 (*p* = 0.0004, r_s_ = −0.516), EBA-181 (*p* = 0.0001, r_s_ = −0.546) as well as for MSP1_19_ (*p* < 0.00001, r_s_ = −0.619). The total IgG responses against EBA-140 and PfRh4 ligands were not associated with levels of parasitaemia (Figure 2).

### IgG1 and IgG3 isotype-specific antibody responses to the EBL and PfRh ligands

IgG1 and IgG3 are the predominant isotypes produced in response to merozoite invasion ligands [4-6], and these isotypes are associated with being cytophilic and mechanistically killing parasitized RBCs by antibody-dependent cell-mediated inhibition (ADCI) [7]. Thus, to gain additional insights in the differential antibody response generated in the Asy and Sym individuals under natural infection conditions, we measured IgG1 and IgG3 responses for PfRh2a, PfRh2b, PfRh5 and EBA-181, for which a higher total IgG response was observed to be negatively correlated with lower parasitaemia (Figure 2). A higher IgG1 seroprevalence was observed in the Asy than the Sym group against EBA-181 (88% *vs*. 24%; *p* = 0.002), PfRh2a (88% *vs.* 38%; *p* = 0.017) and PfRh2b (88% *vs*. 24%; *p* = 0.003). The prevalence of IgG3 responders was higher in Asy than Sym for PfRh2a (75% *vs.* 30%; *p* = 0.039) and PfRh5 (38% *vs.* 3%; *p* = 0.014).

**Figure 3:**
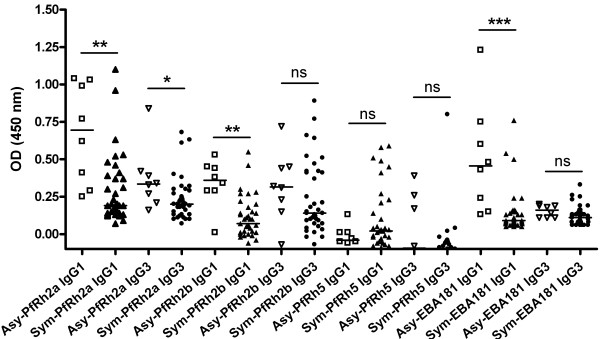
**Comparison of IgG1 and IgG3 isotypes-specific antibody response to EBA-181 and PfRh ligands in asymptomatic and symptomatic Peruvian individuals.** Comparison between the IgG1 and IgG3 responses (as indicated by optical density, OD) against some of the invasion ligands in asymptomatic (Asy) and symptomatic (Sym) Peruvian individuals. Median values are indicated by horizontal bars. Abbreviations: Asy, open symbols; Sym, close symbols; ns, not significant; * *p* < 0.05; ** *p* < 0.01; *** *p* < 0.001.

Differences in IgG1 and IgG3 responses between the Asy \and Sym individuals were also observed (Figure 3). gG1 responses for EBA-181 (median OD 0.46 *vs.* 0.09; *p* = 0.0006) and PfRh2b (median OD 0.36 *vs.* 0.07; *p* = 0.0017) were significantly higher in the Asy than the Sym group. Notably, both IgG1 and IgG3 were dominant in the Asy group for PfRh2a (median IgG1 OD 0.70 *vs.* 0.19; *p* = 0.0018 and median IgG3 OD 0.34 *vs.* 0.2; *p* = 0.0206).

**Figure 4:**
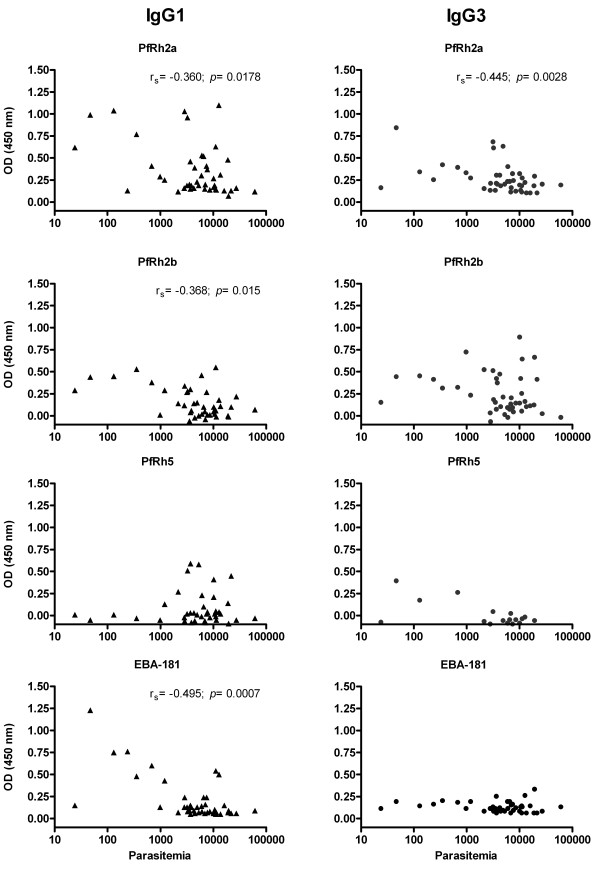
**Association between parasitaemia and the IgG1 and IgG3 antibody response to EBA-181, PfRh2a, PfRh2b and PfRh5 *P. falciparum *ligands.** The parasitaemia was determined by light microscopy and expressed as parasites/μL. Spearman correlation coefficient, abbreviated r_s_*,* and *p* value are shown. Triangles, IgG1 and Circles, IgG3.

Notably, IgG1 and IgG3 levels were also significantly associated with lower parasitaemia (Figure 4). A negative correlation was observed between parasitaemia and the IgG1 levels for EBA-181 (*p* = 0.0007; r_s_ = −0.495), PfRh2a (*p* = 0.0178; r_s_ = −0.360) and PfRh2b (*p* = 0.015; r_s_= −0.368), and the IgG3 levels for PfRh2a (*p* = 0.0028; r_s_ = −0.445).

## Discussion

This study is the first to comprehensively analyse the profile of naturally acquired antibodies against the two major families of *P. falciparum* merozoite ligands in asymptomatic *vs* symptomatic populations living in a malaria hypoendemic area. Sera from *P. falciparum*-infected individuals from the Peruvian Amazon were tested against all PfRh and EBL family members known to be involved in *P. falciparum* invasion. This study’s main finding is that antibody responses against the EBL and PfRh proteins were significantly higher in asymptomatic than symptomatic parasitaemic individuals, suggesting a potential association with the development of clinical immunity. Whether these anti-invasion ligand antibodies directly mediate protective immunity or are simply statistically associated with clinical immunity is beyond the scope of this pilot study. Significant differences in the total IgG responses were observed against EBA-175, EBA-181, PfRh2b and MSP1_19_. IgG1 responses against EBA-181, PfRh2a and PfRh2b were also significantly higher in the asymptomatic individuals. Furthermore, elevated total IgG antibody responses against PfRh1, PfRh2a, PfRh2b, PfRh5, EBA-175, EBA-181 and MSP1_19_ proteins were positively associated with decreased parasitaemia. IgG1 response against EBA-181, PfRh2a and PfRh2b and IgG3 response for PfRh2a were also negatively correlated with parasitaemia. Thus, these results suggest that total and IgG subclass-specific responses to distinct merozoite antigens are significantly associated with protection from high-density parasitaemia and symptomatic malaria.

IgG responses to some but not all EBL antigens has been associated with protection against symptomatic *P. falciparum* in some malaria endemic areas (reviewed in [1]). Naturally acquired antibodies to region II of EBA-175 have been found to increase with age in a naturally exposed population in Kenya and such antibodies were capable of inhibiting binding of EBA-175RII to erythrocytes as a correlate of clinical immunity in this population affected by holoendemic malaria [20]. Nonetheless, significant associations between anti-EBA-175 antibody levels and protection from high-density parasitaemia and clinical disease has been inconsistent among studies [20-23] although experimental studies using sera from rabbits vaccinated with region II of EBA-175 were able to block invasion by > 50% of *P. falciparum* laboratory strains from diverse geographic origin [24,25]. Studies that measure responses to the other EBL antigens in endemic areas outside of Africa are scarcer [5,9,19]. A comparative study by Ford et al., showed that the antibody responses against MSP1_19_ and EBLs (EBA-175, EBA-140, EBA-181) in individuals living in a hypoendemic malaria region of Brazil were much lower thanin individuals living in a hyperendemic area of Cameroon [19].

Immune responses against PfRh invasion ligands have been less studied [4,26,27]. Association between antibody responses against PfRh ligands and outcome of a clinical disease has been reported only for PfRh2a and PfRh2b [4]. Antibody responses against PfRh4, PfRh5 and the common region of PfRh2 were reported for subjects in Kenya without taking into consideration the clinical or parasitological status of the studied individuals [27], and responses against PfRh2b was also evaluated in subjects from Senegal and Tanzania [26]. In children from Papua New Guinea, high levels of total IgG as well as IgG1 and IgG3 antibody responses against the PfRh2a and PfRh2b antigens were found to be strongly associated with protection from symptomatic malaria and high-density parasitaemia. Notably, these antibody responses were similar to or greater than the ones generated against the leading vaccines candidates MSP1_19_, MSP-2 and AMA-1 [4], but, it must be noted, that different regions of the EBAs and the PfRH proteins were studied so it may be difficult to make direct comparisons [4]. Antibody responses against PfPh5 have been found at much lower rates in naturally infected individuals but laboratory studies of anti-PfRh5 antibodies induced by vaccination with recombinant protein appeared to induce strain-transcending growth inhibitory antibodies [25,27]. The focus on PfRh invasion ligands as potential vaccine antigens was augmented by the recent promising invasion inhibitory results obtained using antibodies generated against the combination of EBL and PfRh proteins; EBA-175, PfRh2 and PfRh4 [28], and EBA-175 and PfRh5 [25]. The ability of antibodies against the full length PfRh5 (RH5FL) protein to inhibit the growth of all strains of parasites *in vitro* more effectively than antibodies induced by other vaccine candidates, such as AMA-1 and MSP-1, increased the appeal of using PfRh5 as an anti-blood-stage malaria vaccine [27].

Three studies have previously examined the presence of antibodies against PfRh ligands [4,26,27], but not against PfRh1 in an endemic population. Notably, total IgG response to PfRh1 in the Peruvian population was similar in Sym and Asy individuals based on seroprevalence, but the magnitude of the response was significantly higher in the Asy group and inversely associated with levels of parasitaemia. A high seroprevalence for PfRh2a (75%) regardless of the clinical status (Asy and Sym) was observed in the Peruvian individuals similar to what was reported in children from Papua New Guinea (94%) [4]. However, in Peruvian subjects, significant differences were observed in the prevalence of IgG1 and IgG3 responders in the Asy group (IgG1 88% *vs.* 38% and IgG3 75% *vs.* 30%). In contrast, the response against PfRh2b in the Peruvians (31%) was higher than that reported in individuals with malaria in Senegal (8.9%) and Tanzania (5.6%) and lower than those observed in Papua New Guinea (85%) [4,26]. However, higher total IgG and IgG1 responses against the PfRh2b ligand were associated with low parasitaemia, similar to observed in Papua New Guinea [4]. Seroprevalence for PfRh4 in the Peruvian infected individuals was lower than observed in adults from Kenya (50% *vs.* 70%) [27], and presently it appears that these antigen specific responses are not associated with protection against clinical malaria. In this study only IgG1 and IgG3 subtypes were measured and analysed because these subtypes are associated with F_c_ binding and are potentially associated with functional antibody-dependent cellular cytotoxicity [29,30]. Nonetheless, whether such anti-invasion ligand antibodies might function through ADCI, steric hindering or by another mechanism remains to be determined. Although PfRh5 was suggested to be essential for erythrocyte invasion [31,32] and the target for vaccine-induced antibodies [27], only few individuals from Peru had antibodies against PfRh5 (11%), which is comparable to what was reported in Kenya (16%) [27]. The reason for the lack of anti-PfRh5 antibody in naturally exposed individuals is still unclear. The mechanism by which this essential invasion ligand escapes immune recognition thus requires further investigation.

## Conclusions

Although the contribution of the anti-invasion EBL and/or PfRh ligand antibody responses to protective immunity needs to be confirmed in future studies by functional assays such as growth inhibition assays, the present study strongly suggests that the Peruvian population present in a low transmission setting has a capacity to develop clinical immunity that is likely associated with antibody responses to defined *P. falciparum* invasion ligands. These results are consistent with a potential role for one or more of these invasion ligands as a component of an anti-*P. falciparum* vaccine in the low-transmission areas of the Amazon region, but may argue against a one-size fits all strain-transcending blood stage malaria vaccine.

## Abbreviations

EBL: Erythrocyte binding-like; PfRh: Reticulocyte binding-like; OD: Optical density; Asy: Asymptomatic; Sym: Symptomatic; ADCI: Antibody-dependent cell-mediated inhibition.

## Competing interests

The authors declare that they have no competing interests.

## Authors’ contributions

KT, DG, JMV and SL participated in the design of the study. EV, KT, VN, JB collected the samples. MLP, NT and CL designed or carried out the ELISA studies. EV, MLP, JMV and SL drafted the manuscript. All authors read and approved the final manuscript.

## References

[B1] Tham WH Healer J Cowman AF Erythrocyte and reticulocyte binding-like proteins of *Plasmodium falciparum* Trends Parasitol 2012 28 23 30 10.1016/j.pt.2011.10.002 22178537

[B2] Fowkes FJ Richards JS Simpson JA Beeson JG The relationship between anti-merozoite antibodies and incidence of *Plasmodium falciparum* malaria: a systematic review and meta-analysis PLoS Med 2010 7 e1000218 10.1371/journal.pmed.1000218 20098724 PMC2808214

[B3] Persson KE Erythrocyte invasion and functionally inhibitory antibodies in *Plasmodium falciparum* malaria Acta Trop 2010 114 138 143 10.1016/j.actatropica.2009.05.017 19481996

[B4] Reiling L Richards JS Fowkes FJ Barry AE Triglia T Chokejindachai W Michon P Tavul L Siba PM Cowman AF Mueller I Beeson JG Evidence that the erythrocyte invasion ligand PfRh2 is a target of protective immunity against *Plasmodium falciparum* malaria J Immunol 2010 185 6157 6167 10.4049/jimmunol.1001555 20962255

[B5] Richards JS Stanisic DI Fowkes FJ Tavul L Dabod E Thompson JK Kumar S Chitnis CE Narum DL Michon P Siba PM Cowman AF Mueller I Beeson JG Association between naturally acquired antibodies to erythrocyte-binding antigens of *Plasmodium falciparum* and protection from malaria and high-density parasitemia Clin Infect Dis 2010 51 e50 e60 10.1086/656413 20843207

[B6] Stanisic DI Richards JS McCallum FJ Michon P King CL Schoepflin S Gilson PR Murphy VJ Anders RF Mueller I Beeson JG Immunoglobulin G subclass-specific responses against *Plasmodium falciparum* merozoite antigens are associated with control of parasitemia and protection from symptomatic illness Infect Immun 2009 77 1165 1174 10.1128/IAI.01129-08 19139189 PMC2643653

[B7] Jafarshad A Dziegiel MH Lundquist R Nielsen LK Singh S Druilhe PL A novel antibody-dependent cellular cytotoxicity mechanism involved in defense against malaria requires costimulation of monocytes FcgammaRII and FcgammaRIII J Immunol 2007 178 3099 3106 17312157 10.4049/jimmunol.178.5.3099

[B8] da Silva-Nunes M Moreno M Conn JE Gamboa D Abeles S Vinetz JM Ferreira MU Amazonian malaria: asymptomatic human reservoirs, diagnostic challenges, environmentally driven changes in mosquito vector populations, and the mandate for sustainable control strategies Acta Trop 2012 121 281 291 10.1016/j.actatropica.2011.10.001 22015425 PMC3308722

[B9] Clark EH Silva CJ Weiss GE Li S Padilla C Crompton PD Hernandez JN Branch OH *Plasmodium falciparum* malaria in the Peruvian Amazon, a region of low transmission, is associated with immunologic memory Infect Immun 2012 80 1583 1592 10.1128/IAI.05961-11 22252876 PMC3318420

[B10] Vinetz JM Gilman RH Asymptomatic *Plasmodium* parasitemia and the ecology of malaria transmission Am J Trop Med Hyg 2002 66 639 640 12224566 10.4269/ajtmh.2002.66.639

[B11] Jordan SJ Oliveira AL Hernandez JN Oster RA Chattopadhyay D Branch OH Rayner JC Malaria immunoepidemiology in low transmission: correlation of infecting genotype and immune response to domains of *Plasmodium falciparum* merozoite surface protein 3 Infect Immun 2011 79 2070 2078 10.1128/IAI.01332-10 21383051 PMC3088150

[B12] Alves FP Durlacher RR Menezes MJ Krieger H Silva LH Camargo EP High prevalence of asymptomatic *Plasmodium vivax* and *Plasmodium falciparum* infections in native Amazonian populations Am J Trop Med Hyg 2002 66 641 648 12224567 10.4269/ajtmh.2002.66.641

[B13] Camargo EP Alves F da Silva Pereira LH Symptomless *Plasmodium vivax* infections in native Amazonians Lancet 1999 353 1415 1416 10227233 10.1016/s0140-6736(99)00941-1

[B14] Roshanravan B Kari E Gilman RH Cabrera L Lee E Metcalfe J Calderon M Lescano AG Montenegro SH Calampa C Vinetz JM Endemic malaria in the Peruvian Amazon region of Iquitos Am J Trop Med Hyg 2003 69 45 52 12932096

[B15] Sutton PL Clark EH Silva C Branch OH The *Plasmodium falciparum* merozoite surface protein-1 19 KD antibody response in the Peruvian Amazon predominantly targets the non-allele specific, shared sites of this antigen Malar J 2010 9 3 10.1186/1475-2875-9-3 20047674 PMC2818648

[B16] Mangold KA Manson RU Koay ES Stephens L Regner M Thomson RB Jr Peterson LR Kaul KL Real-time PCR for detection and identification of *Plasmodium* spp J Clin Microbiol 2005 43 2435 2440 10.1128/JCM.43.5.2435-2440.2005 15872277 PMC1153761

[B17] Gaur D Singh S Jiang L Diouf A Miller LH Recombinant *Plasmodium falciparum* reticulocyte homology protein 4 binds to erythrocytes and blocks invasion Proc Natl Acad Sci U S A 2007 104 17789 17794 10.1073/pnas.0708772104 17971435 PMC2077073

[B18] Kaslow DC Hui G Kumar S Expression and antigenicity of *Plasmodium falciparum* major merozoite surface protein (MSP1(19)) variants secreted from Saccharomyces cerevisiae Mol Biochem Parasitol 1994 63 283 289 10.1016/0166-6851(94)90064-7 7516493

[B19] Ford L Lobo CA Rodriguez M Zalis MG Machado RL Rossit AR Cavasini CE Couto AA Enyong PA Lustigman S Differential antibody responses to *Plasmodium falciparum* invasion ligand proteins in individuals living in malaria-endemic areas in Brazil and Cameroon Am J Trop Med Hyg 2007 77 977 983 17984363

[B20] Ohas EA Adams JH Waitumbi JN Orago AS Barbosa A Lanar DE Stoute JA Measurement of antibody levels against region II of the erythrocyte-binding antigen 175 of *Plasmodium falciparum* in an area of malaria holoendemicity in western Kenya Infect Immun 2004 72 735 741 10.1128/IAI.72.2.735-741.2004 14742515 PMC321612

[B21] John CC Moormann AM Pregibon DC Sumba PO McHugh MM Narum DL Lanar DE Schluchter MD Kazura JW Correlation of high levels of antibodies to multiple pre-erythrocytic *Plasmodium falciparum* antigens and protection from infection Am J Trop Med Hyg 2005 73 222 228 16014863

[B22] Okenu DM Riley EM Bickle QD Agomo PU Barbosa A Daugherty JR Lanar DE Conway DJ Analysis of human antibodies to erythrocyte binding antigen 175 of *Plasmodium falciparum* Infect Immun 2000 68 5559 5566 10.1128/IAI.68.10.5559-5566.2000 10992454 PMC101506

[B23] Osier FH Fegan G Polley SD Murungi L Verra F Tetteh KK Lowe B Mwangi T Bull PC Thomas AW Cavanagh DR McBride JS Lanar DE Mackinnon MJ Conway DJ Marsh K Breadth and magnitude of antibody responses to multiple *Plasmodium falciparum* merozoite antigens are associated with protection from clinical malaria Infect Immun 2008 76 2240 2248 10.1128/IAI.01585-07 18316390 PMC2346713

[B24] Jiang L Gaur D Mu J Zhou H Long CA Miller LH Evidence for erythrocyte-binding antigen 175 as a component of a ligand-blocking blood-stage malaria vaccine Proc Natl Acad Sci U S A 2011 108 7553 7558 10.1073/pnas.1104050108 21502513 PMC3088598

[B25] Ord RL Rodriguez M Yamasaki T Takeo S Tsuboi T Lobo CA Targeting sialic acid dependent and independent pathways of invasion in *Plasmodium falciparum* PLoS One 2012 7 e30251 10.1371/journal.pone.0030251 22253925 PMC3257272

[B26] Ahouidi AD Bei AK Neafsey DE Sarr O Volkman S Milner D Cox-Singh J Ferreira MU Ndir O Premji Z Mboup S Duraisingh MT Population genetic analysis of large sequence polymorphisms in *Plasmodium falciparum* blood-stage antigens Infect Genet Evol 2010 10 200 206 10.1016/j.meegid.2009.11.008 19931645 PMC2829370

[B27] Douglas AD Williams AR Illingworth JJ Kamuyu G Biswas S Goodman AL Wyllie DH Crosnier C Miura K Wright GJ Long CA Osier FH Marsh K Turner AV Hill AV Draper SJ The blood-stage malaria antigen PfRH5 is susceptible to vaccine-inducible cross-strain neutralizing antibody Nat Commun 2011 2 601 22186897 10.1038/ncomms1615 PMC3504505

[B28] Lopaticki S Maier AG Thompson J Wilson DW Tham WH Triglia T Gout A Speed TP Beeson JG Healer J Cowman AF Reticulocyte and erythrocyte binding-like proteins function cooperatively in invasion of human erythrocytes by malaria parasites Infect Immun 2011 79 1107 1117 10.1128/IAI.01021-10 21149582 PMC3067488

[B29] Pleass RJ Woof JM Fc receptors and immunity to parasites Trends Parasitol 2001 17 545 551 10.1016/S1471-4922(01)02086-4 11872400

[B30] Roussilhon C Oeuvray C Muller-Graf C Tall A Rogier C Trape JF Theisen M Balde A Perignon JL Druilhe P Long-term clinical protection from *falciparum* malaria is strongly associated with IgG3 antibodies to merozoite surface protein 3 PLoS Med 2007 4 e320 10.1371/journal.pmed.0040320 18001147 PMC2071934

[B31] Baum J Chen L Healer J Lopaticki S Boyle M Triglia T Ehlgen F Ralph SA Beeson JG Cowman AF Reticulocyte-binding protein homologue 5 - an essential adhesin involved in invasion of human erythrocytes by *Plasmodium falciparum* Int J Parasitol 2009 39 371 380 10.1016/j.ijpara.2008.10.006 19000690

[B32] Crosnier C Bustamante LY Bartholdson SJ Bei AK Theron M Uchikawa M Mboup S Ndir O Kwiatkowski DP Duraisingh MT Basigin is a receptor essential for erythrocyte invasion by *Plasmodium falciparum* Nature 2011 480 534 537 22080952 10.1038/nature10606 PMC3245779

